# Brain metastasis from papillary thyroid carcinomas

**DOI:** 10.3892/mco.2013.139

**Published:** 2013-06-26

**Authors:** KYOJI TSUDA, HIDEO TSURUSHIMA, SHINGO TAKANO, KOJI TSUBOI, AKIRA MATSUMURA

**Affiliations:** 1 Department of Neurosurgery, Faculty of Medicine, University of Tsukuba, Tsukuba, Ibaraki 305-8575, Japan; 2 Proton Medical Research Center, University of Tsukuba, Tsukuba, Ibaraki 305-8575, Japan

**Keywords:** brain metastasis, papillary thyroid carcinoma, prognosis

## Abstract

Papillary thyroid carcinoma (PTC) is the most common type of thyroid carcinoma and has a relatively favorable prognosis. PTC brain metastases are rare, occurring in 0.1–5% of cases in previous studies. In the present study, we treated 5 cases of PTC brain metastasis in our institute and retrospectively evaluated these patients. A retrospective database was generated from the patient medical records of our institution for the years between 1976 and 2011. The mean patient age at diagnosis was 64.6 years and the average duration from PTC resection to the detection of a brain metastasis using magnetic resonance imaging (MRI) or computed tomography (CT) was 91.7 months. The patients were treated with various combinations of surgery and radiation therapy. All 5 patients died and the mean overall survival following the diagnosis of a brain metastasis was 9.0 months. One patient succumbed to an intratumoral hemorrhage of the metastatic brain tumor. The remaining patients died following metastasis to other organs. Our findings suggest that PTC brain metastases may occur at the end-stage of patient treatment and result in an unfavorable prognosis. Patients with brain metastases also succumbed to the development of metastases to the fetal organs rather than brain.

## Introduction

Patients with well-differentiated thyroid carcinomas generally have favorable long-term outcomes. Papillary thyroid carcinoma (PTC) is the most common thyroid carcinoma, representing ~80% of the newly diagnosed thyroid carcinomas (1). PTC has a relatively favorable prognosis with a 10-year survival rate of 90–95%. Distant metastases occur less frequently with PTCs compared with other types of thyroid malignancy. When distant metastases are present, the lungs and bones are the most frequently involved organs. By contrast, brain metastases are rare, occurring in 0.1–5% of cases (2). In the present study, we treated 5 cases of PTC brain metastases in our institute and retrospectively evaluated these patients.

## Patients and methods

A retrospective database was generated from the patient medical records of our institution for the years between 1976 and 2011. The patients were histologically diagnosed with PTC and presented with a brain metastasis. The patients with brain metastases that originated from a skull metastasis were excluded.

Of the 5 patients that met our inclusion criteria (Table I), 2 patients were men and 3 patients were women. The median age at diagnosis was 69 years, while the average age was 64.6 years (range, 43–80 years). The pathology of the primary thyroid disease was available for the 5 patients. The average lesion size was 53 mm in diameter (range, 20–110 mm). The average duration from PTC resection to the detection of a brain metastasis on an magnetic resonance imaging (MRI) or computed tomography (CT) scan was 91.7 months (range, 9.6–228.0 months). This study was approved by the Ethics Committee for the University of Tsukuba.

## Results

Multiple brain metastases were observed in 3 of the 5 cases. Lung or mediastinal metastases were detected in 3 cases, and bone metastases were observed in 3 cases. The patients were treated with a varied combination of surgery, stereotactic radiosurgery (SRS), stereotactic radiotherapy (SRT) and ^131^I therapy. Four patients underwent surgery for brain metastases, resulting in a gross total removal (GTR) in 1 patient with a single metastasis. With regards to radiation treatment, 2 patients received SRS and 1 patient underwent SRT. Two patients received ^131^I therapy.

The median Karnofsky performance scores (KPSs) prior and subsequent to the treatments for the brain metastases were 50% (range, 50–90%) and 60% (range, 50–100%), respectively. The patients were deceased at the last follow-up. The mean overall survival following the diagnosis of brain metastases was 9.0±1.414 months (± SD; range, 7–11 months). One patient succumbed to an intratumoral hemorrhage of the metastatic brain tumor, while 3 patients died as a result of lung or mediastinal metastasis progression.

## Discussion

In this retrospective study, the patients diagnosed with PTC brain metastases had an average overall survival of 9.0 months. This survival time is shorter compared with the average overall survival of patients with brain metastases from other thyroid cancers, which has been previously reported to be 4.7–29.2 months (3–8). Chiu *et al*(4) reported that the overall median survival of patients with brain metastases from well-differentiated thyroid cancers was 12.4 months (4.7 months for patients with metastasis from whole thyroid cancer, comprising 11 anaplastic, 32 differentiated and 4 medullary cancers). Of the patients with known causes of death, only 22.2% of the deaths were attributable to brain metastases (7). Our present data regarding the causes of death are similar to the abovementioned data. However, our findings have shown that the prognosis of the 5 patients in this study was worse compared with that in previously published studies (5,6,8,11), with the exception of one study (5) that included a high percentage of anaplastic cases. As shown in Table II, PTC patients who present with other organ metastases have a shorter survival period following the diagnosis of a PTC brain metastasis. Results of the present study may have demonstrated a poorer outcome as our series comprises patients with other organ metastases, particularly metastases to organs of the respiratory system, such as the lung or mediastinum, which might indicate a progressed or advanced disease state. Although most brain metastases were locally controlled, it is hypothesized that other advanced organ metastases or systemic diseases may have caused the death of PTC patients with controlled brain metastases.

Although there is no clearly defined protocol for the management of intracranial metastases from thyroid cancer (9), surgery is generally considered to be the best therapeutic choice for prolonged survival and regression of neurological symptoms (2,3,5,7,9,10). In previous studies, patients undergoing SRS had an overall median survival time of 33–37.4 months (8,11), with GTR being an attractive option for the local control of a solitary or few lesions (9). Hjiyiannakis *et al*(1) noted an apparent benefit of radioactive iodine treatment when uptake scans in the brain were positive, although uptake by cranial metastases is relatively infrequent and has been reported to occur in only 23–25% of cases (7). However, McWilliams *et al*(6) found no evidence to support the use of chemotherapy in patients with brain metastasis from thyroid carcinoma as it was not utilized in any of the studies mentioned previously and no responses were reported in their study cohort. In the present study, the 5 patients who underwent surgery and/or external irradiation therapy had poor prognoses. This result suggested that these therapeutic options did not improve the survival of patients with PTC brain metastases due to systemic advanced disease, but provided good local control of the metastatic brain tumor.

It is generally accepted that patients with metastases from a primary well-differentiated thyroid carcinoma have a favorable prognosis. However, PTC brain metastases should be considered a terminal clinical symptom in PTC and most patients with brain metastases also succumbed to the disease due to the development of metastases to the fetal organs rather than brain, as well as organs of the respiratory system. Therefore, it is important to identify the general condition of the patient and consider the expected prognosis prior to the planning of therapeutic schedules.

## Figures and Tables

**Table I:** Summary of the 5 cases with papillary thyroid cancer brain metastases.

No.	Age (years)	Gender	Duration from T Ca resection to brain meta (months)	No. of brain meta	Lung meta or mediast meta	Bone meta	Removal of brain meta	External Irradiation for brain meta	^131^I therapy	KPS before treatment for brain meta	KPS after treatment for brain meta	Outcome	Survival from brain meta (months)	Cause of death
1	69	M	84	Sig	+	−	P	NP	NP	50	60	Dead	9	Mediast meta
2	60	M	103	Mul	+	+	P	SRS	P	90	90	Dead	9	Lung meta
3	43	F	9.6	Mul	−	+	P	NP	P	70	100	Dead	11	Hemorrhage from brain meta
4	71	F	34	Mul	+	−	P	SRS	NP	50	60	Dead	9	Lung meta
5	80	F	228	Sig	+	+	NP	SRT	NP	50	50	Dead	7	Lung meta

T Ca, thyroid carcinoma; meta, metastasis; mediast, mediastinal; sig, single; mul, multiple; p, performed; NP, not performed; SRS, stereotactic radiosurgery; SRT, stereotactic radiotherapy; KPS, Karnofsky performance score.

**Table II:** Previous studies regarding thyroid cancer brain metastasis.

Author (Refs.)	Year	No. of T Ca	Meta in all T Ca without brain meta	Survival from brain meta (months)	Death due to brain meta	No. of papillary T Ca	Meta in papillary T Ca without brain meta	Survival in papillary T Ca from brain meta (months)
Biswal *et al*(11)	1994	5	4	27.4	0	1	1	6.0
Hjiyiannakis *et al*(1)	1996	6	6	7.8	3	6	6	7.8
Salvati *et al*(8)	2001	12	8	19.8	2	3	2	35.3
McWilliams *et al*(6)	2003	16	3	17.4	2	10	1	23.6
Kim *et al*(5)	2009	9	7	29.2	1	7	6	31.0
Present study	2012	5	5	9.0	1	5	5	9.0

T Ca, thyroid carcinoma; meta, metastasis.
